# Barbarism in New-York

**Published:** 1846-10

**Authors:** 


"*Barbarism in New-York.*"—In the May number of the Western Lancet (which we have received only a few days since,) we find an editorial with the above caption, referring to the imprisonment of Wm. B. Waterman, M. D., for disinterring bodies for anatomical purposes. The editor concludes with the following; "There is one circumstance about this case that we extremely regret, which is, that the physicians of Buffalo permitted Dr. Waterman to remain in prison during the severity of the Winter, for a period of two months, *for the want of bail.*" We feel bound, in behalf of the profession of this city, to state, that the case of Dr. W. was of a somewhat peculiar character. It was generally known, and established on the trial, that his object was not to apply the subjects to his own use in the way of dissections, but to sell them to a medical institution in another state. There was not, therefore, that sympathy felt for him by the profession, and the public, which would doubtless have been the case had the motive been professional improvement instead of pecuniary gain. We know that the Recorder in his sentence was influenced by this consideration. It is due to that functionary, and to the prosecuting attorney, Hon. Geo. P. Barker, to state, that they both represented what has been mentioned, as the only reason which precluded their joining in an application to the Governor for a pardon, very shortly after his being conveyed to prison; and that they consented to make such an application at the expiration of six months. In honor of the liberality of sentiment on this subject which prevails in this city, it should also be said, that the development of the affair, although attended by many circumstances calculated to exasperate the public mind, did not create any considerable excitement, but, on the other hand, a very general disposition to treat it with lenient consideration was manifested. In addition to the enterprise being purely a pecuniary speculation, the fact of there being a partnership with the degraded wretch who was used as State's evidence, by which an extensive, wholesale business was to have been transacted, the latter being the active partner, and the profits to be divided among the concern, served to render the case still more aggravated in its general aspect. It should be added, that Dr. W. had but recently located in this place; had not complied with the conditions required to be recognised as a member of the regular profession, nor had signified his intention of doing so. He was not then, in reality, one of the fraternity of the city.

We have made the foregoing statement as a matter of justice to the medical profession of Buffalo; and we assume to say, that had the case been one of an ordinary character, the medical fraternity, the judicial officers, and the public would have been united in one object, viz, to have disposed of the matter in the easiest possible mode for the accused.

The circumstances of the case, however, repulsive as they may appear in so far as the sordid motives of the individual are concerned, do not in the least affect the inconsistency and barbarism of the laws by which he was punished. The bodies were to have been used for purposes of anatomical knowledge. That knowledge, not only the public welfare and safety demanded, but the laws require under strict penalties. It matters not whether the procurer was to have applied the means for his own scientific improvement, or for the improvement of others. It is enough to show the absurdity and the injustice of the punishment, that the criminal act (as it is legally considered,) tended to no other ultimate end than the public good, and the fulfilment of the requisitions of the law !

